# Modified Biosensor for Cholinesterase Inhibitors with Guinea Green B as the Color Indicator

**DOI:** 10.3390/bios8030081

**Published:** 2018-09-04

**Authors:** Vladimír Pitschmann, Lukáš Matějovský, Martin Lobotka, Jan Dědič, Martin Urban, Michal Dymák

**Affiliations:** 1 Oritest spol. s r.o., Nábřežní 90/4, 150 00 Prague, Czech Republic; pitschmann@oritest.cz (V.P.); lobotka@oritest.cz (M.L.); 2 Faculty of Environmental Technology, University of Chemistry and Technology, Technická 5, 166 28 Prague, Czech Republic; 3 National Institute for Nuclear, Chemical and Biological Protection, Kamenná 71, 262 31 Milín, Czech Republic; dedic@sujchbo.cz (J.D.); urban@sujchbo.cz (M.U.); dymak@sujchbo.cz (M.D.)

**Keywords:** biosensor of cholinesterase inhibitors, chemical warfare agents, enzymatic reaction, Guinea Green B, visual evaluation

## Abstract

Colorimetric biosensors of cholinesterase inhibitors are ideal for fast, reliable, and very simple detection of agents in air, in water, and on surfaces. This paper describes an innovation of the Czech Detehit biosensor, which is based on a biochemical enzymatic reaction visualized by using Ellman’s reagent as a chromogenic indicator. The modification basically consists of a much more distinct color response of the biosensor, attained through optimization of the reaction system by using Guinea Green B as the indicator. The performance of the modified biosensor was verified on the chemical warfare agents (sarin, soman, cyclosarin, and VX) in water. The detection limits ascertained visually (with the naked eye) were about 0.001 µg/mL in water (exposure time 60 s, inhibition efficiency 25%).

## 1. Introduction

Cholinesterase inhibitors adversely affect the transmission of nerve impulses by preventing hydrolytic decomposition of acetylcholine. The accumulation of this neurotransmitter in the area of a synapse is the primary cause of intoxication of the organism. Cholinesterase inhibitors include common organophosphorus and carbamate pesticides, both synthetic and natural substances used in medicine but also as nerve warfare agents [1]. Considerable attention is paid to the detection and determination of cholinesterase inhibitors [2]. In addition to advanced instrumental methods, simple chemical methods are also used in practice, including methods based on color reactions [3]. It is color changes that simple colorimetric detectors are based on, such as indicator papers and strips, detection tubes or various detection kits, and pocket laboratories [4]. Some cholinesterase inhibitors, especially nerve chemical warfare agents, are extremely toxic: for instance, the agents GB (sarin), GD (soman) and GF (cyclosarin) have LCt_50_ by inhalation about 35 mg·min/m^3^, the agent VX even a mere 15 mg·min/m^3^. So, biosensors based on very sensitive enzymatic (cholinesterase) reactions are required for simple detection of the substances in dangerous concentrations [5,6,7,8]. 

The enzymatic (cholinesterase) reaction utilized in simple biosensors is based on color indication of the product of hydrolysis of a suitable substrate. The inhibitor concentration is then proportional to the degree of inhibition of the enzyme and the color change rate. The first biosensors with acetylcholinesterase (AChE) or butyrylcholinesterase (BuChE) contained the substrates acetylcholine and butyrylcholine, which split into choline and the respective acid (acetic or butyric), detectable with a pH-indicator. Recently, however, acetylcholine and butyrylcholine have largely been replaced with acetylthiocholine or butyrylthiocholine, respectively, in which case thiocholine is produced instead of choline. Thiocholine changes the color of redox indicators, such as Ellman’s reagent [9,10,11], 2,6-dichlorophenolindophenol [12] or its analogues [13], or triphenylmethane dyes such as Guinea Green B and Malachite Green [4,14,15]. Chromogenic substrates, e.g., 2,6-dichlorophenolindophenyl acetate [16] and indoxyl acetate [17], which decompose directly on colored products, are frequently used alternatives. A lower reaction rate and poorer availability are the drawbacks.

An example of a biosensor of cholinesterase inhibitors used widely in practice is the Czech Detehit biosensor, which is based on AChE (obtained directly from a pig brain), the substrate acetylthiocholine and Ellman’s reagent as a redox indicator [18]. In spite of its undisputed advantages, this biosensor provides an unclear cut white-yellow color transition, which may cause difficulties especially during visual evaluation in poor light conditions. Earlier, the authors had designed a modification of the Detehit biosensor by using filter paper made from glass nanofibres (as the carrier of the substrate and of Ellman’s reagent), which deepens the intensity of the developing yellow color [19]. They also designed a modified biosensor with the indicator 2,6-dichlorophenolindophenol exhibiting a distinct blue-white color transition [20]. During the further development of this biosensor, the comments, and requirements of potential users (armed forces, rescue corps) were accepted to resist the ambient temperature at around 60 °C for a short time (at least several days). As it turned out, the biosensor with 2,6-dichlorophenolindophenol did not meet this requirement, therefore another, more appropriate indicator was sought. This paper describes an innovated biosensor with Guinea Green B as an indicator providing a distinct green-white color transition, and has increased heat resistance, while maintaining the basic analytical parameters (simple and rapid detection and detection limits).

## 2. Experimental

### 2.1. Chemicals, Materials, and Instruments

Butyrylcholinesterase (BuChE) from horse plasma, butyrylthiocholine iodide (BuTChI), *N*-ethyl-*N*-[4-[[4-[ethyl[(3-sulphophenyl)methyl]amino]phenyl]-phenylmethylene]-2,5-cyclohexadiene-1-ylidene]-3-sulpho-benzenemethane ammonium sodium hydroxide—internal salt (Guinea Green B, C.I. 42085), dextran (all by Sigma-Aldrich, St. Louis, MO, USA), the non-ionic surfactant C12-14 alcohol 7EO, trade name Spolapon 247 (Enaspol, Teplice, Czech Republic), absolute ethanol (Penta, Prague, Czech Republic) and redistilled water were used. A buffer solution at pH 7.3 was prepared using Na_2_HPO_4_·H_2_O and KH_2_PO_4_ (both by Sigma-Aldrich). Physostigmine (Sigma-Aldrich, St. Louis, MO, USA), sarin, soman, cyclosarin and VX (all obtained from the Military Research Institute, Brno, Czech Republic) were used to test the biosensor performance.

The biosensor was prepared by using a white cellulose fabric whose surface density was 173 g/m^2^, cellulose filter paper whose surface density was 85 g/m^2^, and a white plastic pad 0.5 mm thick.

An LMG 173 portable tristimulus colorimeter (Dr. Lange, Dusseldorf, Germany) was used for objective color change measurements. An AB150 instrument (Fisher Scientific, Pardubice, Czech Republic) was employed to measure the pH.

### 2.2. Detector Preparation

**Figure 1:**
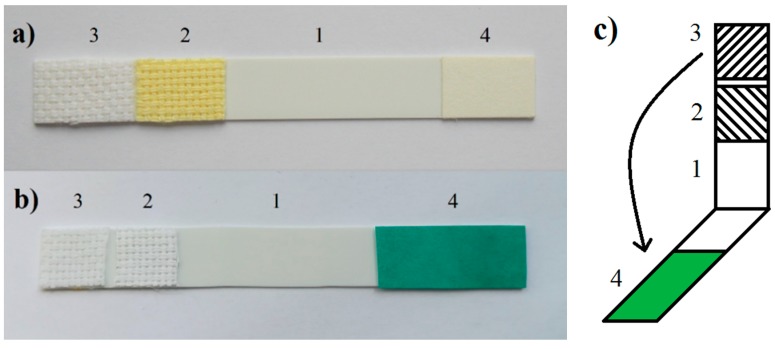
Photograph of (**a**) the Detehit biosensor and (**b**) the modified biosensor: plastic pad 1, etalon 2, fabric with the enzyme-indicator zone 3, paper with the substrate and indicator 4. The layout (**c**) shows how the opposite carriers are folded.

The biosensor was prepared from a plastic pad 10 × 80 mm in size, onto which a carrier with the immobilized enzyme (indicator zone), an etalon, and a carrier impregnated with the substrate and the indicator (Figure 1) were glued. The indicator zone consisted of a white cellulose fabric immersed for 25 min in a solution containing BuChE at a specific activity of 15 nkat/mL, 3.5% (m/m) of dextran and 1.5% (m/m) of a non-ion surfactant in a phosphate buffer solution with pH 7.3. The standard was made from a white cellulose fabric impregnated with a solution containing 9.2% (m/m) of dextran and 5.5% (m/m) of a non-ionic surfactant in a buffer solution pH 7.4. The two impregnated pieces of fabric were dried at 25 °C for 12 h. The working part with the substrate and with the indicator was prepared by immersing the filter paper for 5 min in a solution containing 1.2% of the substrate and 0.2% of Guinea Green B in 50% ethanol. The impregnated paper was dried at 20 °C to 25 °C for 6 h.

### 2.3. Biosensor Testing

The performance of the biosensor was studied in water and in aqueous solutions of selected cholinesterase inhibitors. The working procedure was similar to that used with the Detehit biosensor, with some minor differences matching the design modifications. In step 1, the biosensor (fabric with the enzyme and the etalon) was immersed for 60 s in the sample, analyzed, then removed and rinsed with distilled water. In step 2, the plastic pad was folded (Figure 1c) and the opposite carriers were pressed onto one another so that the paper with the substrate and indicator overlapped both the fabric with the enzyme and the standard. After 120 s, the carriers were separated and the color of the fabric with the enzyme (indicator zones) was observed. The etalon, on which the stable green color remained after the separation of the carriers, served to facilitate the evaluation.

### 2.4. Biosensor Evaluation with the Naked Eye

The inhibitor concentration, which is a function of their inhibitions efficiency (*I*), was determined visually (with the naked eye) based on the relation:

*I* (%) = (1 − T_0_/T) × 100 (1)

where T_0_ is the control time (in seconds) of discoloration of the indicator with the blank (T_0_ = 120 s applies to the proposed system), and T is the time (in seconds) of its discoloration in the presence of the inhibitor. The detection limit corresponded to a concentration that had a 25% inhibitory effect (the indicator was completely decolored in 160 s, 40 s later than the blank). This 25% inhibitory efficacy was selected on the basis of long-term field practice experience in emergency situations that not only required sensitivity but also high detection reliability. The detection limit determined visually (with the naked eye) is not exact, but it is quite common in the field of chemical test methods of analysis [21].

### 2.5. Tristimulus Colorimetry

The proposed method of determining the inhibitors concentration or determination of the detection limit did not require the use of any instrumentation technique (only the time of discoloration was measured). The instrumentation technique (tristimulus colorimeter) was used only to study selected biosensor parameters where the evaluation with the naked eye is difficult or impossible. Tristimulus colorimetry, a.k.a. reflectance colorimetry (spectrophotometry), based on the CIE-L*a*b* color system was employed for the objective measurement of the color changes. In this system, L* represents the neutral brightness axis, a*, the chromatic green-red axis (+a* red, −a* green), and b*, the chromatic blue-yellow axis (+b* yellow, −b* blue). In practice, the color difference Δ*E* was calculated according to the equation

(2) $$\Delta E=\sqrt{\Delta L^{*2}+\Delta a^{*2}+\Delta b^{*2}}$$

is also used, where ΔL*, Δa* and Δb* are the differences between the individual L*, a* and b* values of the standard and the color measured. Differences Δ*E* up to 0.2 are imperceptible with the naked eye, 0.2–0.5 negligible, 0.5–1.5 very small, 1.5–3.0 distinct, 3.3–6.0 very distinct, 6.0–12.0 profound and above 12.0 very profound.

## 3. Results and Discussion

### 3.1. Principle of the Biosensor

**Figure 2:**
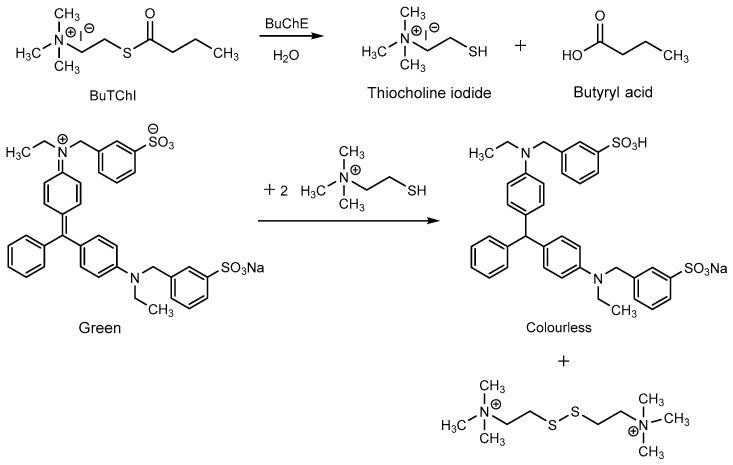

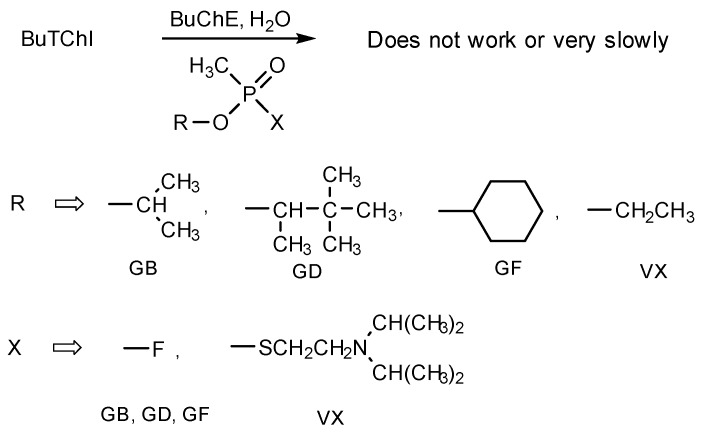
Overall reaction schema.

**Figure 3:**
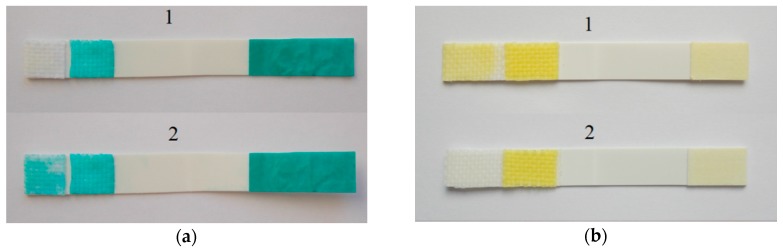
Photograph (**a**): the modified biosensor with a negative result (a1)—blank, and a positive result (a2)—the sample contains physostigmine at a concentration of 10 μg/mL. Both images immediately after separation of the carrier (T_0_ = 120 s). For comparison, photograph (**b**) shows the standard Detehit biosensor with a negative (b1) and positive (b2) result.

The principle of the biosensor consists in BuTChI hydrolysis catalyzed by the BuChE enzyme. The hydrolysis produces thiocholine, which reduces Guinea Green B to its colorless leuco-form. The reaction schema is shown in Figure 2. The practical use of the biosensor was then based on monitoring the kinetics of this enzymatic reaction; i.e., on the rate of discoloration of the indicator zone. Such discoloration proceeds significantly more slowly in the presence of a cholinesterase inhibitor. Figure 3a shows the response of the biosensor (i.e., blank, the indicator zone is completely colorless) and on a sample contaminated by the inhibitor, where the indicator zone remains green after the carriers have been separated. It is evident from a comparison with a standard Detehit biosensor (Figure 3b) that the color change is much more marked with the modified biosensor.

**Figure 4:**
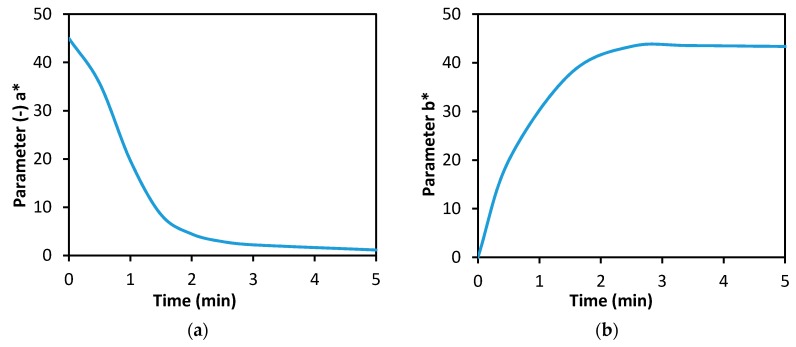
Dependence of the indicator zone color intensity on time (min): (**a**) the modified biosensor (Guinea Green B), (**b**) a standard Detehit biosensor (Ellman’s reagent).

Figure 4a shows one of the basic characteristics of the biosensor designed; i.e., the dynamics of Guinea Green B discoloration during a blank experiment or in the absence of the cholinesterase inhibitor. The curve can be interpreted so that the discoloration of the indicator (assessed visually, with the naked eye) occurred in 2 min (at the moment of separation, the indicator zone was colorless). By way of comparison, Figure 4b shows the dynamics of coloration of the indicator zone of a standard Detehit biosensor.

### 3.2. Effect of the Reaction Conditions

The effect of the BuChE activity on the degree of substrate hydrolysis and the corresponding color effect were also studied. The dependence of the rate of Guinea Green B discoloration on the activity of BuChE manifested itself within the range of 5 to 60 nkat/mL. A further increase in the enzyme activity had virtually no effect on these parameter (values <5 nkat/mL were not tested).

The test results confirmed previous observations [20] that an excess of the substrate over the indicator of 6:1 (*w*/*w*) as a minimum is required for correct performance of the biosensor. It has been found that a lower substrate content will lead to a less marked color contrast and a lower rate of discoloration, which are drawbacks particularly in field conditions. A too high substrate content has to adversely affect the inhibition efficiency, especially of reversible inhibitors such as physostigmine. Based on the relation between the rate of discoloration of the blank (T_0_) and of the sample with the inhibitor (T), an optimum Guinea Green B concentration of 0.2% in the impregnation solution was selected.

The dependence of the biosensor’s performance on the acid-base equilibrium of the medium was monitored as well. The optimum sample pH ranged from 6 to 7. Spontaneous discoloration of Guinea Green B manifested in the alkaline medium at pH > 8, but it did not affect the biosensor function in real time (within 10 min, pH < 10). The fact that increasing pH is accompanied by acceleration of the hydrolysis of inhibitors such as sarin and VX [22] must, of course, also be taken into account in practical settings.

### 3.3. Detection Limits

**Figure 5:**
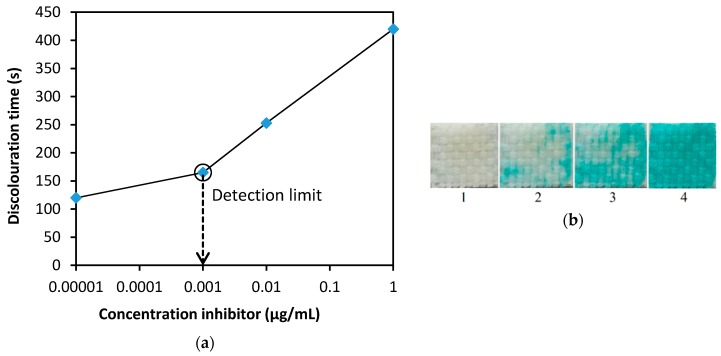
(**a**) Dependence of discoloration time on concentration—the average values of GB, GD, GF and VX were used to construct the graph, see Table 1, (**b**) dependence of color intensity (appearance of indicator zone) on inhibitor concentration at the control time T_0_ = 120 s (1—blank, 2—0.001 µg/mL, 3—0.01 μg/mL, 4—1 μg/mL). Note: the curve in figure is based on a fit of data points.

The detection limits were determined on samples of the most important nerve agents (GB, GD, GF, VX). During tests under comparable experimental conditions (i.e., enzyme activity in the impregnation solution 15 nkat/mL), exposure time 60 s, enzyme-substrate contact time 120 s (T_0_), were around 0.001 µg/mL. More detailed data on the test results are provided in Table 1. These values comply with military standards. For instance, NATO Standard [23] permits the maximum concentration of nerve agents in drinking water of 0.012 µg/mL for consumption of 5 L per day (i.e., a daily dose of 60 µg). Figure 5a shows the dependence of the time of discoloration of the indicator zone on the concentration of inhibitors; this graph can be used to estimate the current concentration. It can be seen that concentrations of less than 0.001 µg/mL can be detected by the proposed biosensor, but in this case, a blank control should be performed. Figure 5b shows the appearance of the indicator zone at the control time (T_0_ = 120 s), depending on the concentration of the tested nerve agents.

### 3.4. Interferences

The selectivity of methods based on the cholinesterase reaction is rather well-known. It is based on the chemical properties of the enzyme, substrate, and indicator used. In general, detection affects significant reductants, strong oxidants, and acid and alkaline agents. The high content of organic solvents also has a negative effect. Most of these interfering effects can be limited to a certain extent by the appropriate design of the biosensor. It has been experimentally verified that the detection by the proposed modified biosensor did not affects the presence of bicarbonates, carbonates, phosphates, sulfates, chlorides, nitrites, and nitrates (all Na salts) to a concentration of 1000 mg/L. Since the biosensor contains a redox indicator, the effect of some strong reducing substances has also been verified. Sodium bisulphite and sodium sulfide, both at a concentration of 1000 mg/L, induced rapid, resp. very fast discoloration of the indicator. At a concentration of 25 mg/L their effect was no longer observed. In conclusion, under such conditions, high concentrations of agents do not occur. These results only document the high chemical resistance of the biosensor.

### 3.5. Stability

**Figure 6:**
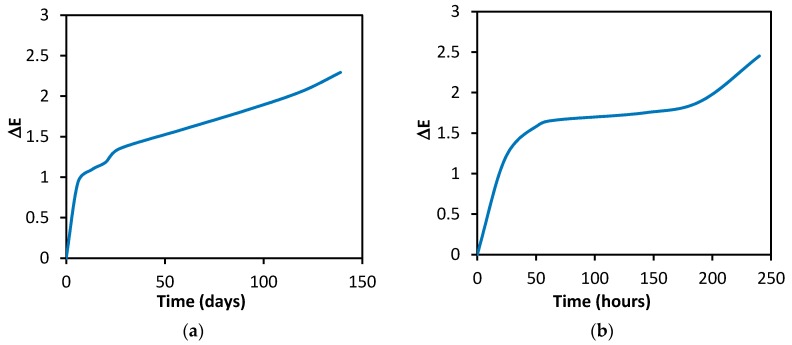
Spontaneous decrease in the color intensity of the filter paper with the substrate and Guinea Green B. Stored (**a**) in normal laboratory conditions, and (**b**) in a drying room at 60 °C.

**Figure 7:**
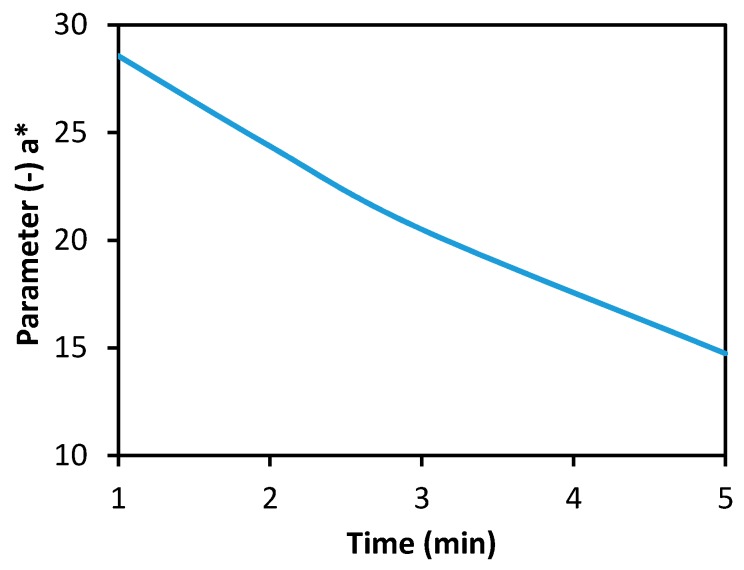
Performance of the modified biosensor with the filter paper impregnated with the substrate and the indicator after exposure to a thermal load of 60 °C for 240 h. The sample contained 10 µg/mL of physostigmine, conditions of measurement: exposure 60 s, carriers separated after 60 s.

Samples of the fabric with the immobilized enzyme were stored in normal laboratory conditions. The enzyme activity did not change significantly during 18 months of storage in a hermetically sealed container and at room temperature. The substrate and the indicator on filter paper stored in identical conditions remained stable and functional for 12 months as a minimum. Both of the substances on the filter paper were relatively stable even during long-term storage in normal laboratory conditions in a non-hermetic environment (in an unsealed polyethylene bag). As shown in Figure 6a, the largest decrease in the intensity of the original green color occurred during the initial phase of storage. The discoloration was registered on the tristimulus colorimeter; it was not observed visually (with the naked eye). Based on comments and requests from potential users, the resistance of the filter paper with the substrate and the indicator to a thermal load of 60 °C was also examined. Figure 6b demonstrates that the color degradation was about 10 times faster for Δ*E* = 1 and even about 25 times faster for Δ*E* = 1.5 than under normal conditions. The test with physostigmine at a concentration of 10 µg/mL confirmed that the filter paper with the substrate and the indicator remained functional (although to a limited extent) even in 240 h of storage at 60 °C (Figure 7). Filter paper with substrate and 2,6-dichlorophenolindophenol [20], tested under the same conditions, lost its function after several hours. The stability of the enzyme on the carrier was satisfactory, after 10 days of storage at 60 °C, activity was decreased by 27%.

## 4. Conclusions

The modified Detehit biosensor with immobilized and stabilized BuChE, with a BuTChI substrate and Guinea Green B as the indicator, allows fast, simple and robust detection of cholinesterase inhibitors. The performance of the biosensor in water was verified on the carbamate inhibitor physostigmine and on the major military organophosphorus inhibitors sarin, soman, cyclosarin, and VX. The detection limits for the mentioned organophosphates were around 0.001 µg/mL, which match those of the Detehit biosensor as well as other technical means of detection based on the enzymatic method. The main advantage of the innovated biosensor is its clear-cut green-white color transition, which enables easy visual evaluation (with the naked eye). This is beneficial especially when using the biosensor in the field and/or in poor light conditions. Another advantage of the biosensor is its resistance to high ambient temperatures. Unlike the earlier 2,6-dichlorophenolindophenol biosensor, the new Guinea Green B biosensor does not lose functionality, even with a limitation, even after 10 days exposed to 60 °C.

## Figures and Tables

**Table 1:** Experimental data acquired during the determination of the inhibitor concentrations: the indicator zone discoloration time and the corresponding inhibition efficiency (I) of major nerve agents. The data in the table are mean values (rounded to tens) of 4 measurements, the results were independently evaluated by at least three observers.

Sample	0.001 µg/mL		0.01 µg/mL		1 µg/mL	
	Discoloration Time (s)	*I* (%)	Discoloration Time (s)	*I* (%)	Discoloration Time (s)	*I* (%)
GB	170 (±10)	29.4	290 (±20)	58.6	>420	>71.5
GD	160 (±10)	25.0	240 (±20)	50.0	>420	>71.5
GF	160 (±10)	25.0	180 (±20)	33.4	>420	>71.5
VX	170 (±10)	29.4	300 (±20)	60.0	>420	>71.5
Average	165 (±10)	27.2	252.5 (±20)	50.5	>420	>71.5
